# Commentary: Designing healthcare for human use: human factors and practical considerations for the translational process

**DOI:** 10.3389/frhs.2023.1188114

**Published:** 2023-06-16

**Authors:** Bo Kim

**Affiliations:** ^1^ Center for Healthcare Organization and Implementation Research, VA Boston Healthcare System, Boston, MA, United States; ^2^ Department of Psychiatry, Harvard Medical School, Boston, MA, United States

**Keywords:** commentary, translation, human factors, human-centered design, conceptual frameworks, adaptations, implementation strategies, implementation science

A commentary on Designing healthcare for human use: human factors and practical considerations for the translational process by Edwards III GF, Zagarese V, Tulk Jesso S, Jesso M, Harden SM and Parker SH. (2023). *Front Health Serv*. 2:981450. doi: 10.3389/frhs.2022.981450

## 1. Introduction

Human factors’ influence on health care practices is of heightened interest to the field (1), driving numerous efforts that pursue higher quality care by accounting for human behaviors, abilities, and limitations (2). Given implementation science's focus on achieving better care through promoting the uptake and sustained use of evidence-based interventions (3), and especially as human-centered design approaches to implementation are being increasingly embraced (4), an explicit connection between implementation science and human factors begs to be conceptualized. Edwards et al. offer a timely Perspective article that articulates this very connection, using implementation case examples to demonstrate how human factors interact with the design and implementation of evidence-based interventions (5). Notably, the article also provides a helpful list of specific human factors considerations to enhance an intervention's use, fidelity, and sustainability, presented in alignment with the widely-used Reach Effectiveness Adoption Implementation Maintenance [RE-AIM; (6)] framework's Adoption, Implementation, and Maintenance domains, respectively. The purpose of this commentary is to further contextualize the article's notions of human factors by discussing specific examples of their expected relevance to additional implementation science concepts, approaches, and foci, in the hopes of fueling continued discourse on integrating human factors considerations into implementation endeavors.

## 2. Human factors considerations for implementation frameworks

By outlining human factors considerations per RE-AIM's Adoption, Implementation, and Maintenance domains, Edwards et al.'s article illustrates the potential for human factors considerations to work complementarily even with other implementation frameworks beyond RE-AIM. Especially for frameworks that are understood to be broad in their domain definitions (to be widely applicable across various interventions and implementation contexts), human factors considerations can help specify what the frameworks delineate as factors that influence implementation. For instance, the Integrated Promoting Action on Research Implementation in Health Services [i-PARIHS; (7)] framework consists of four domains (Innovation, Recipients, Context, and Facilitation), using which it posits that successful implementation of an innovation and its sustained use by recipients in a context are enabled by facilitation. The human factors considerations outlined in the article (e.g.: “In what ways does the intervention fit within the user's current work and workflow?” “How are individuals trained to complete the steps in an intervention?”) directly align to i-PARIHS domains (e.g., Innovation and Recipients, respectively). Hence, an i-PARIHS-guided implementation effort can straightforwardly extend its use of i-PARIHS to specifically include relevant human factors considerations per domain. For example, the implementation effort's Innovation- and Recipients-related key informant interviews can include questions about the intervention's fit with current workflows and involved individuals’ training status, respectively.

## 3. Human factors considerations for implementation adaptations

Edwards et al.'s article emphasizes the importance of human factors considerations particularly for adapting an intervention to fit the involved individuals' capabilities that they can exercise, given the system(s) in which they operate. This emphasis suggests that human factors considerations can meaningfully contribute to planning and evaluating adaptations that are made as the intervention is implemented. For instance, Iterative Decision-making for Evaluation of Adaptations [IDEA; (8)] is a tool that can be used to methodically decide whether and how to proceed with making adaptations to an intervention. A major decision point in IDEA involves assessing whether there is a need for adaptation based on existing knowledge (e.g., published data, input from involved individuals). Incorporating human factors considerations directly into this decision point can be one way to help ensure that human factors are accounted for in making decisions regarding adaptations. Namely, in seeking the knowledge upon which to make the decision, published data can be examined and individuals' input can be sought specifically regarding, for example, the extent to which the intervention fits with current workflows and individuals’ training status.

## 4. Human factors considerations for implementation strategies

Many of the human factors considerations outlined in Edwards et al.'s article focus on human behaviors, abilities, and limitations as they relate to an intervention being implemented. Warranting further attention is how the considerations apply to devising the implementation strategy (or strategies) to be employed, for promoting the uptake and sustained use of the intervention. One way to incorporate human factors considerations into strategy design could be to augment Proctor et al.'s framework for specifying and reporting implementation strategies (9) with human factors considerations. Specifically, the Justification domain of the framework, defined as the “empirical, theoretical, or pragmatic justification for the choice of implementation strategies,” can ask explicitly for human factors-related justifications (e.g., how the strategies account for current workflows and involved individuals' training status).

## 5. Discussion

Edwards et al.'s Perspective article provides essential conceptual building blocks using which the integration of human factors and implementation science can be pursued by the field going forward. This commentary aims to expand on the implications of the article by describing three potential ways in which the human factors considerations outlined in the article can be synergistic with existing ways in which frameworks, adaptations, and strategies are regarded in implementation science. Building from the article and this discussion, future works can systematically assess the impact of bringing human factors and implementation science together, studying the effectiveness, as well as costs and benefits, of incorporating human factors considerations into designing and implementing evidence-based interventions. In parallel with these assessments, also needed are efforts to more clearly delineate the overlaps and distinctions between human factors considerations and notions such as acceptability, appropriateness, and feasibility that have established definitions within implementation science (10). Especially given Edwards et al.'s explanation of incorporating the human factors perspective into implementation studies as “a minor but pivotal shift” to how most implementation studies are currently undertaken, this delineation is important to accurately understand the unique contributions of both the article and the human factors perspective more generally to implementation science.
